# Erythrocyte (red blood cell) dataset in thalassemia case

**DOI:** 10.1016/j.dib.2022.107886

**Published:** 2022-02-02

**Authors:** Dyah Aruming Tyas, Tri Ratnaningsih, Agus Harjoko, Sri Hartati

**Affiliations:** a Department of Computer Science and Electronics, Universitas Gadjah Mada, Yogyakarta, Indonesia; b Department of Clinical Pathology and Laboratory Medicine, Faculty of Medicine, Nursing and Public Health, Universitas Gadjah Mada, Yogyakarta, Indonesia

**Keywords:** Erythrocyte, RBC, Thalassemia, Classification, Peripheral blood smears

## Abstract

Red blood cell (RBC) dataset was obtained from four thalassemia peripheral blood smears and a healthy peripheral blood smear. The dataset contains 7108 images of individual red blood cells for nine cell types. The first process is image acquisition, which is the process of retrieving microscopic image data from peripheral blood smears through a Olympus CX21 microscope using an Optilab advance plus camera. Laboratory assistants helped obtain ideal erythrocyte images. We provide peripheral blood smear from four thalassemia patients in the ThalassemiaPBS dataset. After image acquisition, the image is resized from 4100 × 3075 pixels to 800 × 600 pixels to reduce the computing load in the next stage. We extracted the green color component (green channel) of the RGB image and used it in the next process. We chose the green channel because it is not affected by variations in color and brightness. Furthermore, the segmentation stage is carried out to obtain an object in the form of a single red blood cell. After that, the object can be classified according to the type of red blood cell. This dataset can become an opportunity for international researchers to develop the classification method for red blood cells.

## Specifications Table

**Table:** 

Subject	Computer Science Applications
Specific subject area	Red blood cell classification in anemia case using computational tools and automatic learning methods.
Type of data	Image
How data were acquired	The RGB images were taken by Optilab advance plus camera and taken from Olympus CX21 microscope. Then, The RGB images processed in MATLAB for segmentation to obtain individual cell images. The cell images obtained were labelled and stored by the clinical pathologists.
Data format	.png for grayscale
Parameters for data collection	The peripheral blood smear images were taken with 1000x total magnification of the oil immersion objective lens (100x) when combining with a 10x eyepiece. We used peripheral blood smear from thalassemia patients, and healthy individuals. There are nine types of cells collected in this dataset: elliptocyte cell, pencil cell, teardrop cell, acanthocyte cell, stomatocyte cell, target cell, spherocyte Hypochromic cell, Normal cell. In this dataset, We combine elliptocytes cell and ovalocytes cell in elliptocyte cell.
Description of data collection	The thalassemia peripheral blood smear images (ThalassemiaPBS dataset) were taken by Optilab advance plus camera and taken from Olympus CX21 microscope. We took the peripheral blood smear images with 1000x total magnification of the oil immersion objective lens (100×) combined with a 10× eyepiece. The original image resolution is 4100 × 3075 pixels (RGB images). The image is resized to 800 × 600 pixels to reduce the computing load in the next stage. We extracted the green color component (green channel) of the RGB image because it is not affected by variations in color and brightness. Furthermore, the segmentation stage is carried out to obtain an object in the form of a single red blood cell. Then the manual sorting is done to classify the single red blood cell images by type. There are nine types of cells collected in RBCdataset: elliptocyte cell, pencil cell, tear drop cell, acanthocyte cell, stomatocyte cell, target cell, spherocyte, Hypochromic cell, Normal cell. The resolution of single red blood cell varies widely, depending on the size of the cell.
Data source location	Institution: Department of Clinical Pathology and Laboratory Medicine, Faculty of Medicine, Nursing and Public Health, Universitas Gadjah Mada (UGM) City/Town/Region: Yogyakarta Country: Indonesia Latitude and longitude (and GPS coordinates, if possible) for collected samples/data: −7.768428746252419, 110.37418824602783
Data accessibility	ThalassemiaPBS: https://simpan.ugm.ac.id/s/yiDp0Voqt6LCV30 or https://data.mendeley.com/datasets/gd9ysj73jd/1 Tyas, Dyah Aruming; Ratnaningsih, Tri; Harjoko, Agus; Hartati, Sri (2022), “ThalassemiaPBS”, Mendeley Data, V1, doi: 10.17632/gd9ysj73jd.1 RBCdataset (single Erythrocyte): https://simpan.ugm.ac.id/s/hdgN3G4lNkVAvjR or https://data.mendeley.com/datasets/rfdz6wfzn4/1 Tyas, Dyah Aruming; Ratnaningsih, Tri; Harjoko, Agus; Hartati, Sri (2022), “RBCdataset”, Mendeley Data, V1, doi: 10.17632/rfdz6wfzn4.1 Universitas Gadjah Mada will grant a persistent identifier to our dataset. If there any further question, you can send an email to aharjoko@ugm.ac.id or dyah.aruming.t@ugm.ac.id to request the dataset.
Related research article	Tyas, D. A., Hartati, S., Harjoko, A., & Ratnaningsih, T. (2020). Morphological, Texture, and Color Feature Analysis for Erythrocyte Classification in Thalassemia Cases. IEEE Access, 8, 69,849–69,860. https://doi.org/10.1109/ACCESS.2020.2983155

## Value of the Data

• The ThalassemiaPBS dataset is a peripheral blood smear images collected from thalassemia patients. At present, it is infrequent for a public dataset to relate to microscopic images of thalassemia's peripheral blood smear. Therefore, this dataset will be a source of data for computer application researchers related to thalassemia.
• The RBCdataset is a single erythrocyte dataset collected from thalassemia's peripheral blood smear. This dataset will be a source of data for computer application researchers related to RBC classification.
• Researchers interested in solving red blood cell segmentation cases, especially in thalassemia cases, can use the ThalassemiaPBS dataset.
  Researchers interested in solving red blood cell classification cases, especially in thalassemia cases, can use this dataset.
• Presented data can be used to develop an RBC classification system or as additional data in the system development process.
• Researchers can further analyze this data to obtain the most representative features for each cell type.
• This data can become an opportunity for international researchers to develop a work support system for the pathologist.

## 1 Data Description

**Fig. 1:**
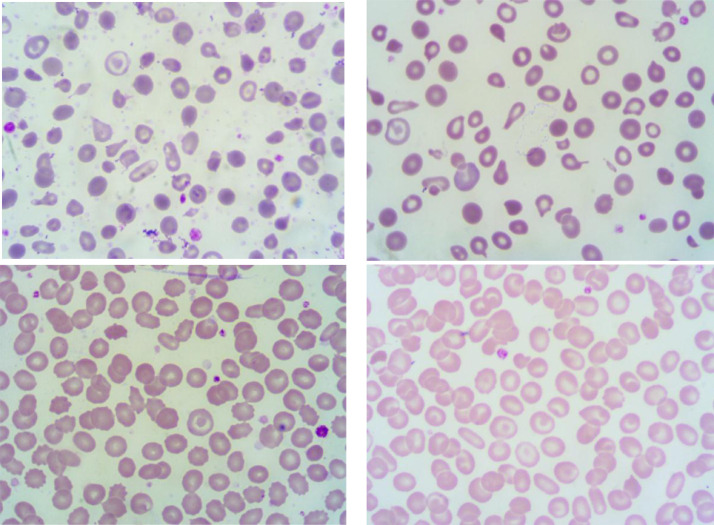
Peripheral Blood Smears Image of Thalassemia Patients.

The peripheral blood smear (PBS) images dataset comes from four thalassemia patients. PBS images were taken by Optilab advance plus camera (8 MP) and taken from Olympus CX21 microscope. We took the peripheral blood smear images with 1000x total magnification of the oil immersion objective lens (100x) combined with a 10x eyepiece. The original image resolution is 4100 × 3075 pixels. We provide 20 images of each patient for this dataset. The example of ThalassemiaPBS images is shown in Fig. 1.

The red blood cell dataset consists of nine cell types with a total of 7108 cells. The images format is a .png in a grayscale image. The size varies according to the size of the cell dimensions. Image cells are grouped into nine cell types by a clinical pathologist from the Clinical Pathology Laboratory of the Faculty of Medicine, Public Health and Nursing, Universitas Gadjah Mada, Indonesia, as shown in Table 1. An example image for each cell type is shown in Table 2. In RBCdataset, the elliptocyte type consists of elliptocyte and ovalocyte. Elliptocytes are cells with the long axis is more than twice the short axis, while ovalocytes are cells with the long axis is less than twice the short axis. Hypochromic is a cell that has a central pallor greater than one-third of the RBC diameter. Whereas the acantocyte cells include burr cells in them.

**Table 1:** Type and number of cells.

Cell Type	Total of Images by type	%
Elliptocyte cell (elliptocyte, ovalocyte)	1211	17.04
Pencil cell	24	0.34
Tear drop cell	2076	29.02
Acanthocyte cell	354	4.98
Stomatocyte cell	382	5.37
Target cell	851	11.97
Spherocyte	562	7,91
Hypochromic cell	222	3.12
Normal cell	1426	20.06
Total	7108	100

**Table 2:** Sample of red blood cell image for each cell type [1].

## 2 Experimental Design, Materials and Methods

The red blood cell images derived from 4 thalassemia patients peripheral blood smear and a healthy peripheral blood smear. The procedure of peripheral blood smear preparation was carried out according to the guidelines in [2] using wedge technique:

1. A blood drop (approximately 2 to 3 mm in diameter) of EDTA anticoagulated blood is placed at one end of the slide. The pusher slide, held securely in the dominant hand at an angle of about 30 to 45° (Fig. 2, A), is drawn back into the drop of blood, and the blood is allowed to spread over the entire width of the slide (Fig. 2, B). It is then quickly and smoothly pushed forward onto the end of the slide to create a wedge film (Fig. 2, C).

**Fig. 2:**
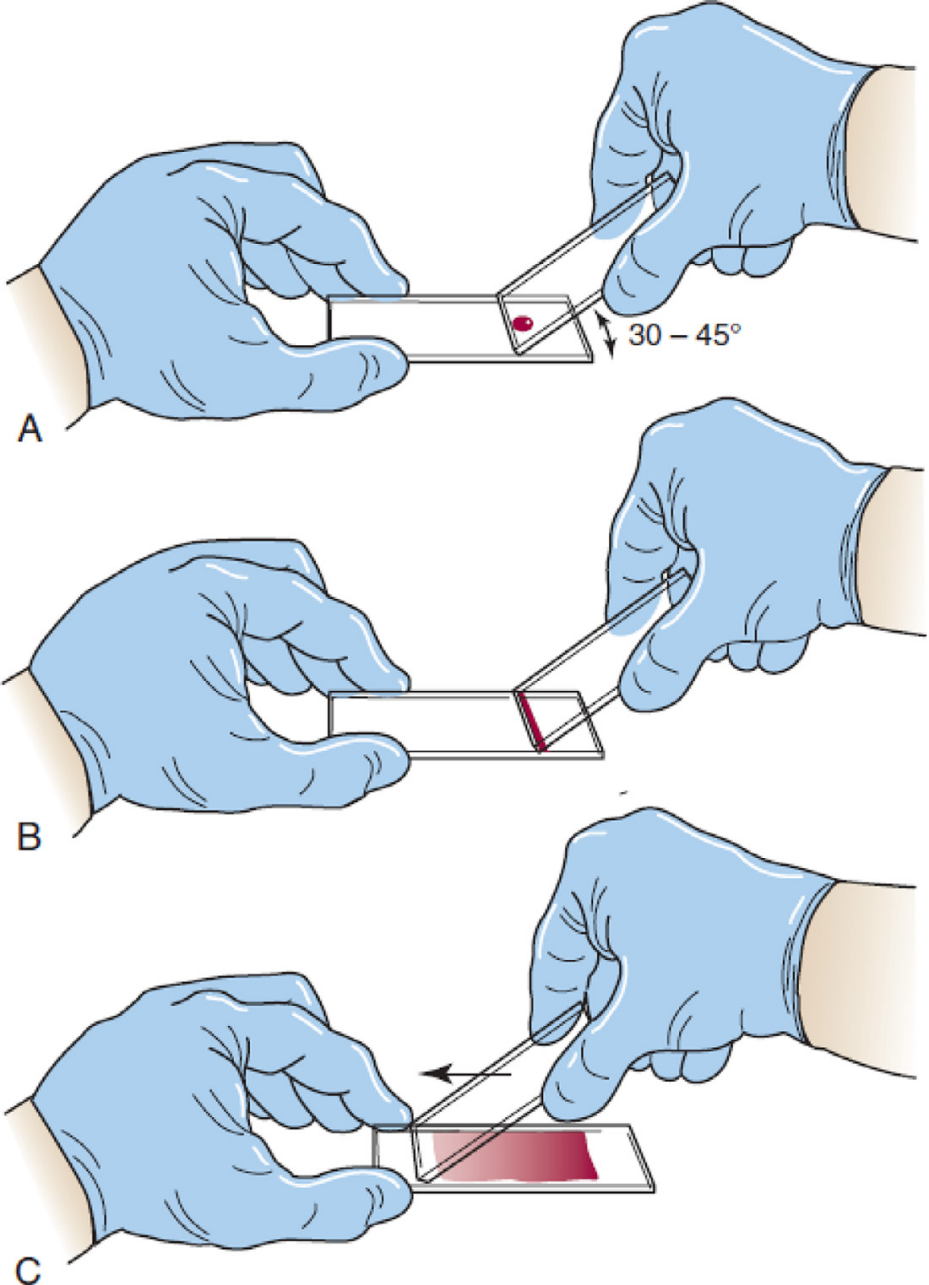
Wedge technique of making a peripheral blood smear [2].
2. After the film preparation method, before staining, all blood films should be dried as quickly as possible to avoid drying artifacts.
3. The slide is placed on the shelf, the film side facing up. Pure Wright stain or Wright-Giemsa stain (Romanowsky stain) is used. Wright stains can be filtered before use or poured directly from the bottle through the filter onto a slide. It is essential to flood the slide completely. The stain must remain on the slide for at least 1 - 3 min for the cells to adhere to the glass. Then approximately the same amount of buffer is added to the slide. Surface tension allows the very little buffer to flow. The mixture was allowed to remain on the slide for 3 min.
4. When staining is complete, the slide is rinsed with a steady but gentle stream of neutral pH water, the back of the slide is cleaned to remove stain residue, and the slide is air-dried in a vertical position.

**Fig. 3:**
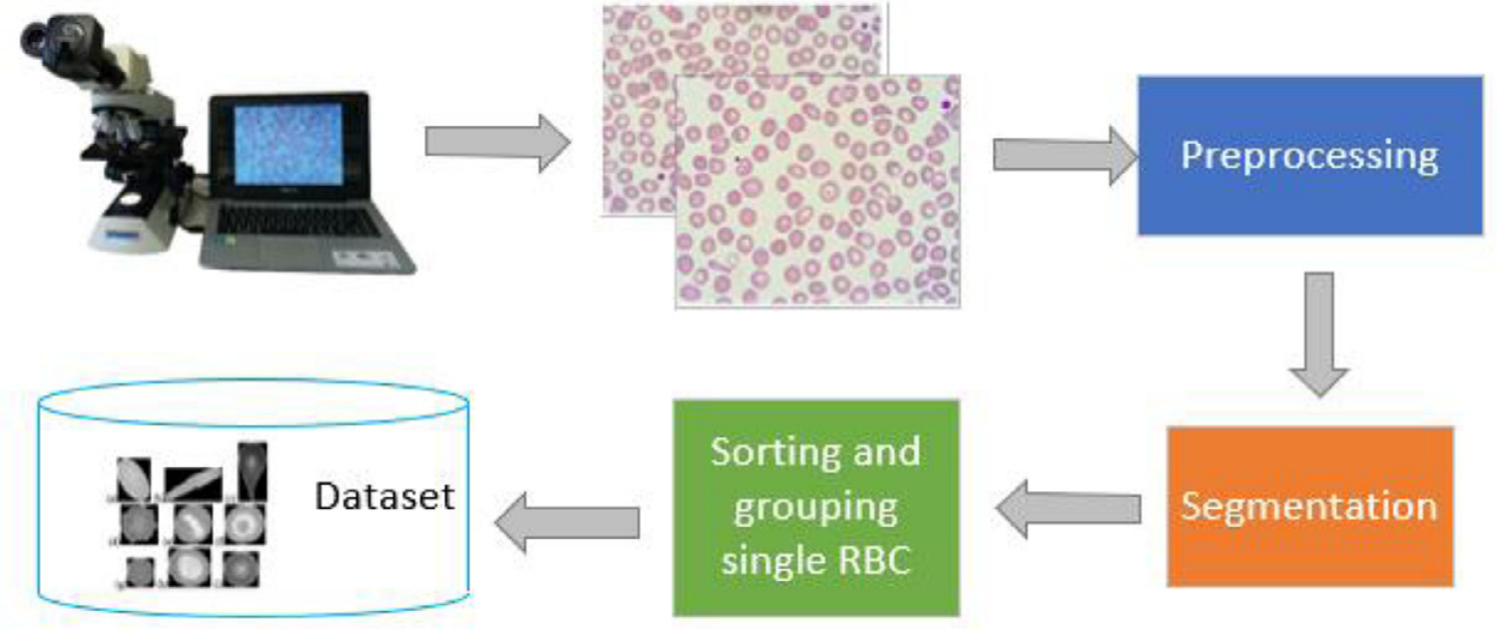
The whole process of dataset collection.

**Fig. 4:**
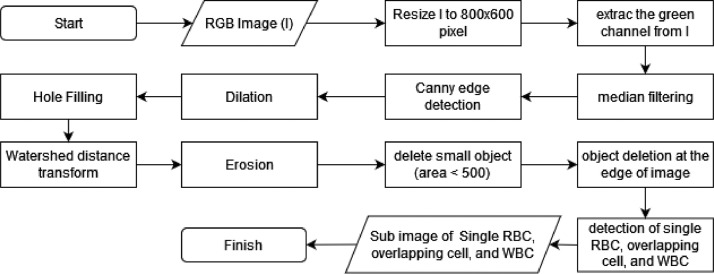
The method used in pre-processing and segmentation stages.

Furthermore, digital image retrieval using a microscope and additional camera. As shown in Fig. 3, the following process is carried out according to the stages in the study of Tyas et al., [1]. In the preprocessing step, the image is resized from 4100 × 3075 pixels to 800 × 600 pixels to reduce the computing load in the next stage. Then, we used the green channel of the image and used it for the following process. The method used in preprocessing and segmentation stages is shown in Fig. 4. The segmentation stage is carried out to obtain red blood cell candidates. Median filtering, canny edge detection, dilation, and hole filling were used. WDT operation is used to separate the overlapping erythrocytes. Then erosion is applied, followed by removing small objects with an area below 500 pixels. We chose 500 pixels because the minimum value of the area feature obtained in the dataset was 526 pixels, so the closest value was determined, 500 pixels. The cells at the edge of the image are deleted because they have an incomplete cell shape.

**Fig. 5:**
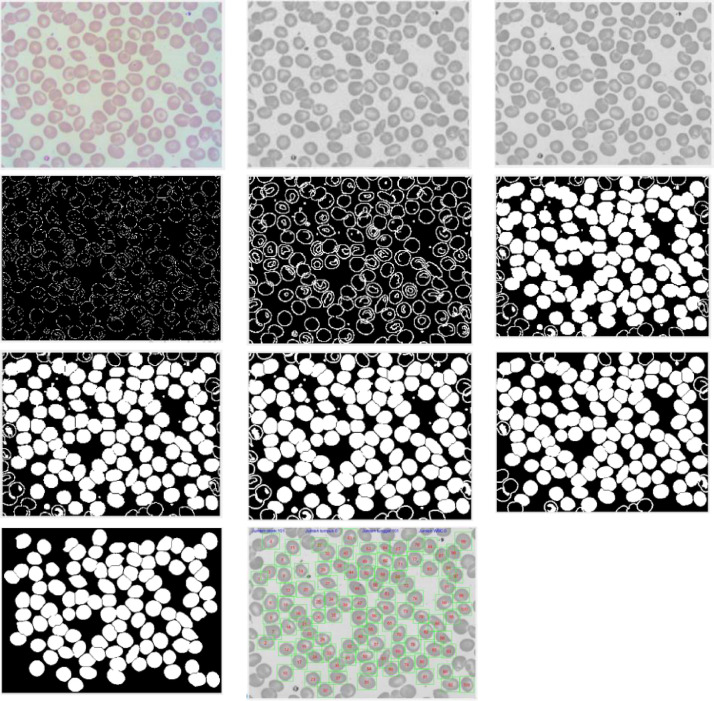
Sample of result image for every method used in pre-processing and segmentation stage. Sequentially in the first row: sample image (RGB), green channel image, median filtering result. The second line in sequence: canny edge detection result, dilation result, hole filling result. The third row in sequence: watershed result, erosion result, deletion of small object result. The fourth row in sequence: object deletion at the edge of image and image result of cell detection.

Next, the detection of single erythrocytes, overlapping erythrocytes, and white blood cells (WBC) was done. The detection process is carried out on all cells in the visual field image based on thresholding to detect the objects. We used area, color intensity, and eccentricity parameters in the thresholding process. The sample of image results for every method used in preprocessing and segmentation stage is shown in Fig. 5. Finally, the clinical pathologist carried out the sorting and grouping process to determine the cells used as a dataset. A dataset of red blood cells with nine cell types was obtained from this stage. This process follows the nomenclature from ICSH [3].

## CRediT authorship contribution statement

**Dyah Aruming Tyas:** Conceptualization, Methodology, Software, Writing – original draft, Formal analysis. **Tri Ratnaningsih:** Supervision, Resources, Validation. **Agus Harjoko:** Supervision, Writing – review & editing. **Sri Hartati:** Supervision, Writing – review & editing.

## Declaration of Competing Interest

The authors declare that they have no known competing financial interests or personal relationships which have or could be perceived to have influenced the work reported in this article.
